# The function of T cells in immune thrombocytopenia

**DOI:** 10.3389/fimmu.2025.1499014

**Published:** 2025-02-21

**Authors:** Siyuan Bu, Min Liu, Lu Yang, Pamela Lee, Heather Miller, Chan-Sik Park, Maria Byazrova, Alexander Filatov, Kamel Benlagha, Timo Gaber, Frank Buttgereit, Quan Gong, Zhimin Zhai, Chaohong Liu

**Affiliations:** ^1^ Department of Pathogen Biology, School of Basic Medicine, Tongji Medical College and State Key Laboratory for Diagnosis and Treatment of Severe Zoonotic Infectious Diseases, Huazhong University of Science and Technology, Wuhan, Hubei, China; ^2^ Tongji Hospital, Tongji Medical College, Huazhong University of Science and Technology, Wuhan, China; ^3^ Department of Rheumatology and Clinical Immunology, Charité – Universitätsmedizin Berlin, Corporate Member of Freie Universität Berlin and Humboldt-Universität Zu Berlin, Berlin, Germany; ^4^ German Rheumatism Research Centre (DRFZ) Berlin, Institute of the Leibniz Association, Berlin, Germany; ^5^ Department of Paediatrics and Adolescent Medicine, Li Ka Shing Faculty of Medicine, The University of Hong Kong, Hong Kong, Hong Kong SAR, China; ^6^ Cytek Biosciences, R&D Clinical Reagents, Fremont, CA, United States; ^7^ Department of Pathology, Asan Medical Center, University of Ulsan College of Medicine, Seoul, Republic of Korea; ^8^ Laboratory of Immunochemistry, National Research Center Institute of Immunology, Federal Medical Biological Agency of Russia, Moscow, Russia; ^9^ Institut de Recherche Saint-Louis, Université de Paris, Paris, France; ^10^ Department of Immunology, School of Medicine, Yangtze University, Jingzhou, Hubei, China; ^11^ Department of Hematology, The Second Hospital of Anhui Medical University, Hefei, China

**Keywords:** immune thrombocytopenia, CD4+T cell, CD8 + T cell, genetic factors, T cell

## Abstract

Immune thrombocytopenia (ITP) is an autoimmune disease, characterized by increased bleeding due to a reduced platelet count. The pathogenesis of ITP is very complex and involves autoantibody production and T-cell-mediated immune abnormalities. An imbalance of effector and regulatory CD4^+^ T cells and the breach of tolerance primarily cause ITP, leading to the dysfunctional development of autoreactive Th cells (including Th1, Th2, and Th17 cells) and Tregs. The loss of auto-platelet antigen tolerance in ITP results in autoantibody- and cytotoxic T-cell-mediated platelet clearance. T-cell-related genetic risk factors significantly influence the development and progression of this disease. New therapies targeting T cells have emerged as potentially effective cures for this disease. This review summarizes the role of T cells in ITP.

## Introduction

Platelets are circulating anucleate blood cells with a short lifespan produced by shedding long-branching cytoplasmic protrusions called proplatelets from bone marrow resident megakaryocytes and eliminated in the spleen and liver (1). Thrombopoiesis is driven by liver-derived thrombopoietin stimulating thrombopoietin receptors in megakaryocytes to form proplatelets. This process can be further enhanced via pro-inflammatory IL-6 increasing blood thrombopoietin levels. Platelets’ physiological functions include the process of hemostasis and coagulation, which are crucial for maintaining vascular integrity by interacting with the vascular wall and play a role in wound healing and angiogenesis. Moreover, they store and release many plasma factors, such as growth factors, vascular tone regulators, and cytokines. These factors are intricately linked to physiological inflammatory responses, infections, and immune-mediated inflammatory diseases (2, 3). Platelets can survive in the peripheral blood circulation for 7-10 days before they are cleared by phagocytes in the reticuloendothelial system (4). Healthy adults produce approximately 10^11^ platelets per day, which can increase 10-fold upon demand (5).

Immune thrombocytopenia (ITP) is an autoimmune disease characterized by a low platelet count (less than 100,000/µL) affecting both adults and children with a prevalence of about 1/10000 in adults (6). Depending on the duration, ITP can be classified as newly diagnosed (<3 months), persistent (3-12 months), and chronic (>12 months) (7). Adults usually present with a chronic course of the disease, whereas children usually have an acute onset (8). The reduction of platelets causes impaired hemostasis and uncontrolled bleeding (9). Some patients are asymptomatic, but about 2/3 of patients have bleeding of the skin (petechiae and purpura) or the oral and nasal mucosa, while others have hematuria or menorrhagia. Most adult ITP patients have a favorable prognosis and a relatively low mortality rate (10), while a tiny percentage of patients experience severe and fatal hemorrhage (e.g., intracranial hemorrhage) (11). Despite the favorable prognosis of most patients, a decrease in health-related quality of life (HRQOL) is common (7). A study carried out in the United States in 2008 demonstrated that HRQOL was much lower in ITP adults than in the normal population. HRQOL is also relatively low in ITP adults compared to patients with hypertension, arthritis, or cancer (12). Therefore, the pathogenesis of ITP needs to be clarified to identify effective preventive and therapeutic methods to improve patients’ cure rate and HRQOL.

The first study on the pathogenesis of ITP was conducted in 1915 when Frank et al. hypothesized that ITP was due to the inhibition of platelet production, which was possibly caused by the abnormality of splenocytes. In 1916, Kaznelson et al. first reported the efficacy of splenectomy in ITP patients, providing further evidence for the increased destruction of platelets caused by splenocyte abnormalities. In 1951, William Harrington drew 500 ml of whole blood from a patient with active chronic ITP and injected himself with it. Two hours after the injection, his platelet count dropped from 800 × 10^9^/L to 25 × 10^9^/L. After 24 hours the number gradually dropped to zero but recovered in the following days (13). Several subsequent studies have confirmed that increased platelet destruction is a hallmark of ITP. It is still unclear how the complex pathophysiologic mechanism causing the decreased platelet level in ITP works. Early studies suggested that autoantibodies, which specifically bind to platelet antigens, trigger platelet destruction through activation of the complement system (14). In about 50% of ITP patients, antiplatelet antibodies targeting platelet surface glycoproteins (GPs) were detectable (15). The IgG type, especially IgG1, makes up the majority of these autoantibodies and accounts for about 77%. In addition to IgG, small amounts of IgA and IgM are also present. These autoantibodies mainly bind to GPIb/IX or GPIIb/IIIa. In 70-80% of patients, they are specific for GPIIb/IIIa, and in 20-40%, they are specific for GPIb/IX; however, in some patients, they are against both antigens (16). Megakaryocytes are obviously targeted by antiplatelet antibodies because they express the same GPs as platelets, which results in decreased platelet production and impaired thrombopoiesis (17).

Another mechanism involved is Fcγ receptor (FcγR)-mediated phagocytosis, which contributes significantly to platelet destruction (18). In ITP, monocytes express higher levels of FcγRI and FCγRIIa/FCγRIIb ratio. These changes are associated with an enhanced capacity for phagocyte antigens.

**Figure 1:**
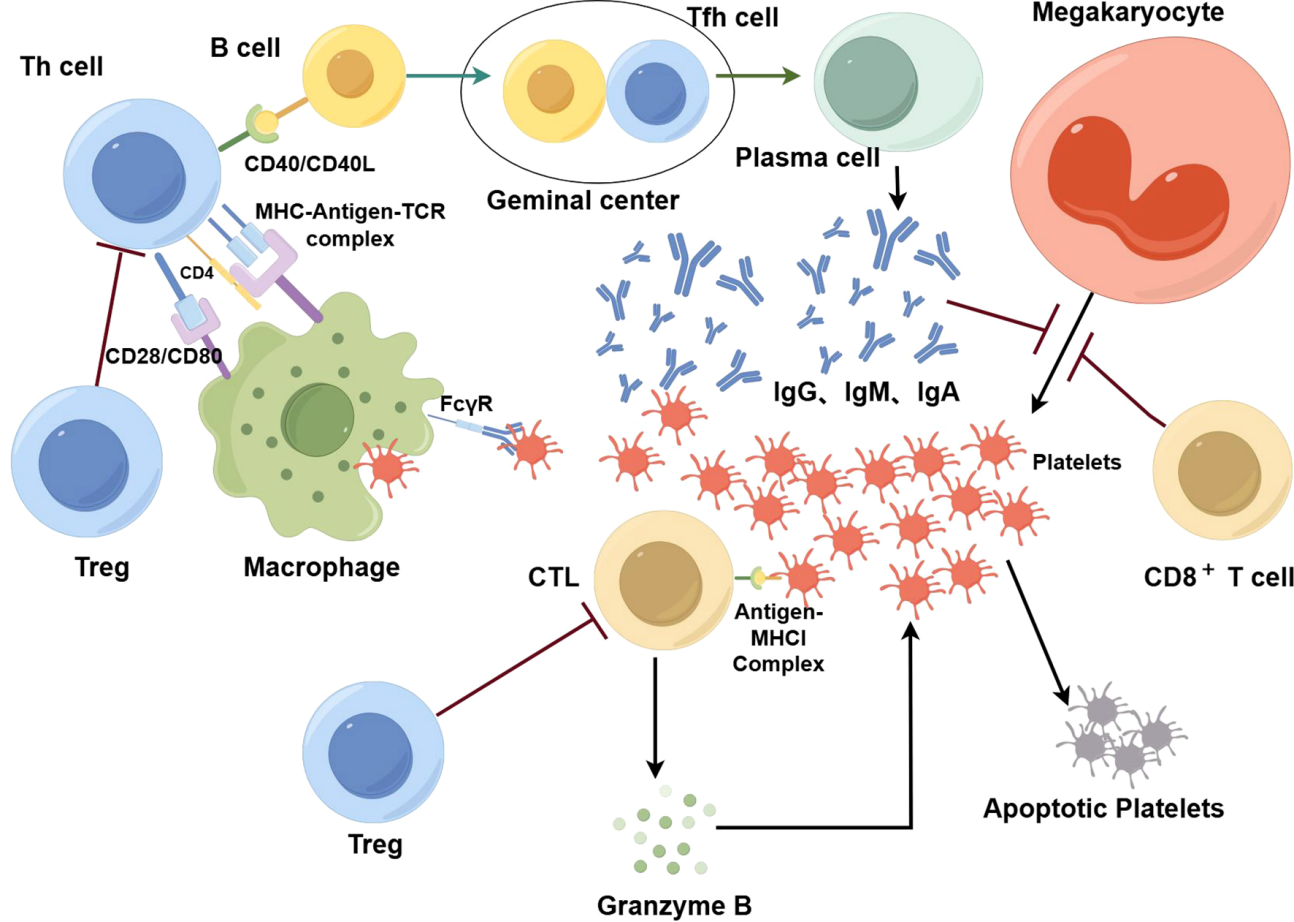
In ITP, T cells play an important role in the destruction of platelets. Platelets are phagocytized and processed by macrophages, where they bind to MHC molecules and are presented as antigen-MHC complexes on the cell surface. These complexes attach to receptors on the surface of Th cells, triggering their activation; once activated, Th cells stimulate B cells. With the additional help of Tfh cells, activated B cells differentiate into plasma cells that produce antibodies against platelets. These antibodies are predominantly IgG with some IgA and IgM. After being bound by antibodies, platelets are phagocytized by macrophages via FcγR. In addition, specific antibodies have an inhibitory effect on the production of platelets by megakaryocytes. CTLs also play an important role in the destruction of platelets. Platelets can express antigen-MHCI complexes on their surface, which in turn are recognized by CTLs. Then, CTLs secrete perforin, granzyme A, and granzyme B which consequently destroy platelets via cytotoxicity. CD8^+^ T cells also suppress megakaryocyte apoptosis and then increase the number of dysfunctional megakaryocytes, leading to suppression of megakaryocyte maturation and platelet production. Tregs inhibit the function of effector T cells so that the destruction of platelets is controlled. When the number and function of T cells are dysregulated, the self-tolerance mechanism of platelets can be disrupted, the production of antiplatelet autoantibodies can increase, and macrophage phagocytosis can be enhanced. Moreover, CTL-mediated cytotoxicity intensifies, resulting in greater platelet destruction.

However, recently, attention has been given to the T cell compartments. As a major part of adaptive immunity, T cells are critical for immune defense and self-tolerance. Naïve CD4^+^ T cells can become various types of cells, including T helper (Th) cells, T follicular helper (Tfh) cells, and regulatory T cells (Tregs). Each type of T cell has unique characteristics and functions and is important for the proper immune response (19). It is therefore not surprising that T cell abnormalities or imbalances in different T cell subsets are closely related to various diseases, such as autoimmune diseases, including ITP (20). ITP is mainly connected to the imbalance of CD4^+^ T cells and cytotoxic T cell (CTL)/CD8^+^ T cells-mediated platelet clearance (21) (**Figure 1**) (By Figdraw). In addition, recent studies have demonstrated that ITP is a multifactorial disease, and is linked to environmental and genetic risk factors. Gaining knowledge on genetic factors that are associated with T cells in ITP can be powerful for the diagnosis, prevention, and treatment of ITP.

## Dysregulation of CD4^+^ T cells in ITP

Over the years, more and more research has shown that dysregulated CD4^+^ T-cell-mediated immunity is critical in the pathogenesis of ITP. The main contributor to ITP is the imbalance between Th1 and Th2 cells. In addition, Tregs function and numbers are inhibited, while Tfh cells, which provide T cells with the ability to facilitate the production of antibodies, including those raised against platelets, are elevated in the spleen and peripheral blood (22).

### Th1/Th2 elevation

T cells are classified into different subpopulations according to the cytokines they secrete, which include Th1 cells and Th2 cells among others (23). Upon activation, naïve T cells can develop into Th1 cells if IL-12 and IFN-γ are present, after which they secrete IFN-γ and IL-2. This process is controlled by the transcription factor T-box (T-bet). Th2 cells are induced by IL-4 and secrete cytokines like IL-4, IL-5, IL-9, IL-10, and IL-13, a process controlled by the transcription factor GATA-3 (24). Th1 cells mainly respond to intracellular pathogens and are associated with organ-specific autoimmunity. Th2 cells, on the other hand, play an important role in the immune response against extracellular parasites and are involved in anaphylactic reactions (25). The balance between Th1/Th2 is very important in the immune response and is dysregulated in many autoimmune diseases. In ITP patients, the Th1/Th2 ratio has significantly increased compared to healthy subjects. This suggests that either Th1 polarization is favored or Th2 polarization is attenuated in ITP. Furthermore, an elevated Th1/Th2 ratio is negatively linked to peripheral platelet counts (26). Accordingly, the ratio of T-bet/GATA-3 expression in peripheral blood mononuclear cells (PBMCs) and splenocytes in patients is increased (27). After treatment with prednisone or splenectomy, the Th1/Th2 ratio decreased and was close to normal range. Furthermore, cytokine production in chronic ITP patients exhibited a clear Th1-polarized cytokine profile at both mRNA and protein levels (28). This pathological Th1 cytokine profile, including IL-2 and IFN-γ, induces a pro-inflammation immune response, that promotes and enhances macrophage-mediated phagocytosis as well as cytotoxicity causing the abnormal destruction of platelets in ITP patients (23, 29). A study published in 1991 has shown that increased IL-2 production stimulates T-cell activation, which in turn activates B cells to produce antibodies and also autoantibodies against platelets in ITP (30). IFN-γ is the major cytokine used to distinguish Th1 cells from other CD4^+^ subpopulations. Its main role is to promote inflammation as a reaction to intracellular pathogens (31). The increase of IFN-γ by Th1 polarization upregulates FcγR expression and downregulates inhibitory FcγRIIB expression, which lowers the macrophage activation threshold thereby enhancing phagocytic capacity. The enhanced capability to phagocyte and process proteins, pathogens, dead cells, and cell debris contributes to the MHCII-mediated presentation of short peptides of foreign- and self-antigens [e.g., GPIIb/IIIa (32)] to CD4^+^ T cells without the proper control by Tregs. Subsequently, B cells are activated and produce pathogenic anti-platelet antibodies (33). The Th1 phenotype is favored in this process, leading to an elevated Th1/Th2 ratio. Accordingly, a higher Th1/Th2 ratio correlates with a higher level of platelet-associated IgG (28).

### Reduced number and impaired function of Tregs

Regulatory T cells (Tregs) maintain immune homeostasis and peripheral self-tolerance by reducing the excessive effector T cell response, preventing autoreactive effector T cells from exerting their unwanted function, and thus inhibiting autoimmune diseases such as ITP. To this end, Tregs express cytotoxic T-lymphocyte-associated antigen-4 (CTLA-4) and glucocorticoid-induced tumor necrosis factor receptor (GITR) and mainly secrete cytokines like IL-10, IL-35, and TGF-β.

In ITP patients, the number and frequency of Tregs in peripheral blood and bone marrow are lower than those in healthy subjects (34). Tregs from ITP patients exhibit lower amounts of FOXP3, which was also observed in a murine model of ITP (35). ITP patients have significantly reduced mRNA levels of CTLA-4 and GITR, and the secretion and production of IL-10 and TGF-β are less compared to normal individuals (36). Moreover, Treg numbers are inversely associated with the severity of ITP syndromes, and the number of Tregs increases greatly during complete remission, especially after splenectomy (37); thus, the decrease of Tregs is particularly pronounced in patients with refractory ITP (8). Overall, the inhibitory function of Tregs and their number are diminished in individuals with ITP. When the number of Tregs is reduced and their function decreases, Tregs do not effectively inhibit the activation of auto-reactive T-cells in ITP, which in turn causes an increase in antiplatelet autoantibodies, i.e., resulting in decreased self-tolerance and enhanced autoimmune responses (34). There may be different explanations for the mechanism contributing to an altered Treg number and function in ITP. IL-35 is an immunosuppressive cytokine that can both increase the production of Tregs and cause Tregs to secrete immunomodulatory factors (e.g., IL-35 and IL-10). The plasma concentration of IL-35 tends to be lower in patients when ITP is in the active phase. This reduction is even more remarkable in the bone marrow, suggesting that the abnormality of Tregs in ITP may be related to the decrease in IL-35 (38). Results from another study have suggested a new potential mechanism for the lack of Tregs in the peripheral blood in ITP, such as central thymic retention of Tregs, which depletes peripheral tolerance mechanisms and permits an immune response to process antiplatelet antibodies (39). The exact mechanism responsible for the change in the number and function of Tregs in ITP is still unclear, but understanding the significant role Tregs play in ITP could prove valuable for therapeutic purposes.

Maintaining the homeostasis of T cells requires the Jak-STAT and PI3K-AKT pathways, which seem to be over-activated in ITP. Low-dose anti-cancer drug decitabine, which is a DNA methyltransferase inhibitor that induces cell cycle arrest and apoptosis, has been demonstrated to prevent the activation of STAT3, promoting the development of Tregs and enhancing their immunosuppressive function. A clinical trial demonstrated that for patients receiving low-dose decitabine, the number and function of Tregs are significantly improved compared to pre-treatment, while Th1 and Th17 cells are suppressed; therefore, these patients’ clinical symptoms were improved significantly (40). Histone deacetylase inhibitors (HDACi) can increase the number and function of Tregs and have anti-inflammatory and immunomodulatory effects (41). Another study has shown that in ITP models, low-dose HDACi (chidamide) can alleviate the disease by increasing the number and function of Tregs and platelet counts (42).

### The abnormality of Th17/Treg

In addition to overacting Th1 cells and limiting Treg response, Th17 cells opposing Tregs have implications for the pathogenesis of ITP (6). In 2005, a specific type of Th cells capable of secreting IL-17, Th17 cells, was first discovered (43). Th17 cells secrete different pro-inflammatory cytokines, including the eponymous cytokines IL-17 and IL-21. These cytokines are important. For example, IL-17A, a member of the IL-17 cytokine family, is strongly associated with asthma (44). While infections caused by bacteria and fungi are significantly prevented by IL-17, IL-21 connects the innate and adaptive immune responses, with their imbalance being associated with autoimmune diseases and cancer (45). For example, IL-21 and IL-17 levels are elevated in patients with systemic lupus erythematosus (SLE) due to the expansion of Th17 cells and the reduction of Tregs (46). Th17 cells and Tregs have opposite functions in the immune response. Due to their contrary roles, the ratio of Th17/Treg influences the outcome of the immune response. Therefore, the balance between Th17 cells and Tregs is a crucial factor in autoimmune diseases and is tightly regulated via signaling cascades, transcriptional and translational regulation, and metabolic reprogramming (47).

Retinoic acid receptor-related orphan receptor γ (ROR γ) is crucial for the differentiation of Th17 cells. In ITP, the level of secreted IL-21 and the ROR γ mRNA expression are both elevated. Also, the ratios of Th17/Treg and ROR γ/Foxp3 are increased (48). Compared with mild ITP and healthy controls, patients with severe ITP have a more pronounced increase in the Th17/Treg ratio, which has an inverse relationship to the platelet count (49). The imbalance of Th17 cells and Treg cells is closely associated with cancers, pathogen infections, and autoimmune diseases such as ITP (50). Factors affecting the Th17/Treg ratio include T-cell surface proteins, cell metabolism, miRNAs, and cytokines, which may contribute to ITP.

T-cell receptor (TCR) complexes and co-stimulatory receptors are T cell surface proteins that take part in both activation and suppression of T cell functions. After recognizing and binding to antigen-bound MHC molecules, TCRs activate different downstream signaling pathways through immunoreceptor tyrosine-based activation motifs (ITAMs), which in turn guide T cell differentiation into different subtypes with different metabolic programs depending on the co-stimulatory signals and metabolic program (e.g., CD28, CTLA4, cytokines). Therefore, TCR and co-stimulatory signaling control the balance of Th17/Treg (51). The mechanisms that regulate TCR-mediated T cell activation and activity also contribute to the balance of Th17/Treg. One example is the PD-1/PD-L1 interaction pathway. Activated T cells mainly express PD-1, while PD-L1 expression in antigen-presenting cells (APCs) is constitutively low. PD-1 binds to PD-L1, which activates the signaling pathway for apoptosis and suppression of effector T cells, preventing collateral damage induced by an over-active immune response. This negative feedback mechanism keeps the immune response in check and maintains immune homeostasis, but if the negative feedback does not function properly, it can cause autoimmune diseases. In patients with chronic ITP, the levels of PD-1 and PD-L1 appear lower compared to healthy subjects, as reported by Zhong et al. (52). In another study, the percentage of PD-1^+^CD4^+^ T cells and PD-L1^+^DC cells was found to be higher than that of healthy controls by flow cytometry. Still, there was a significant imbalance of CD4^+^ T cells. At the same time, the PD-1/PD-L1 pathway was inhibited (53). In conclusion, abnormalities in T cell surface proteins certainly break the balance of Th17/Treg, which in turn causes ITP.

A second regulatory mechanism involves miRNAs. These short, single-stranded non-coding RNAs have approximately 19-25 nucleotides and regulate gene expression in different ways, including inhibition of protein translation and alteration of mRNA stability. Some miRNAs are dysregulated in ITP and thus, significantly affect the expression of genes that regulate Th17/Treg homeostasis by controlling the differentiation of Th17 and/or Treg cells ultimately causing a Th17/Treg imbalance (54, 55). Aberrant expression of miRNAs may take part in the onset and progression of ITP, suggesting that they can be useful biomarkers for the diagnosis and treatment of ITP. Indeed, differentially expressed miRNAs can serve as tools to improve ITP diagnosis as demonstrated by Zuo et al. (56). Additionally, inflammatory cytokines, especially TGF-β, IL-6, IL-21, IL-23, and IL-1β, are also significant for regulating Th17/Treg homeostasis. Although Th17 and Treg cells have opposite functions, they are produced by common progenitor cells (naïve CD4^+^ T cells). Therefore, Th17 cells and Tregs have the same TGF-β pathway. In naïve CD4^+^ T cells, TGF-β induces Foxp3 and ROR γ to be expressed, after which naïve cells begin to develop into Th17 and Treg cells (57). With the help of IL-6, IL-1β, IL-21, and IL-23, Th17 cell differentiation from naïve CD4^+^ T cells is induced by TGF-β. However, without these cytokines, TGF-β suppresses the activity of ROR γ while promoting the expression of Foxp3, which causes naïve cells to differentiate into Tregs (58). Unlike that in healthy controls, the expression of TGF-β tends to be lower in chronic ITP patients. This abnormality is negatively related to platelet counts and is important for maintaining disease activity in chronic ITP (59, 60).

Current studies show that the balance of Th17/Treg reflects the regulatory conditions of the immune system and inflammatory state, suggesting that it may be a better indicator of the severity and prognosis of ITP disease (61). Moreover, since the imbalance of Th17/Treg can cause ITP, regaining the Th17/Treg homeostasis can be a new way to treat ITP. After four days of high-dose dexamethasone (HD-DXM) treatment, chronic ITP patients had an increase in Treg cells (62). Additionally, the concentration of TGF-β greatly increased after treatment with HD-DXM. Therefore, HD-DXM can increase Treg cells and thus improve symptoms during chronic ITP flare-ups (63). It is worth mentioning that a study has shown that IL-10 expression was reduced in patients who were ineffective on DXM therapy, while IL-17 was consistently expressed. This result can be used as a biomarker for corticosteroid refractory ITP (64).

### Increase in Th22 cells

Th22 cells were first reported in 2009. Th22 cells belong to the T helper cell that participates in adaptive immunity by secreting IL-22. As a transcriptional regulator, Aryl hydrocarbon Receptor (AhR) is crucial for Th22 cells (65). In preliminary studies on patients with ITP, it was found that the concentration of IL-22 in the plasma was increased, which is closely connected with the count of Th1 and Th22 cells, but Th17 cells also secrete large amounts of IL-22, making it uncertain whether the increased level of IL-22 in ITP is mainly caused by Th1 and Th22 cells. However, further research has revealed that circulating Th17 cells are statistically similar between patients and healthy controls. What is more, in patients with active ITP, the increased level of IL-22 and the proportion of Th17 cells are not closely correlated. This suggests that the elevated IL-22 level is mainly caused by the increased number of Th1 and Th22 cells, which are the main T-cell subpopulations secreting IL-22, rather than Th17 cells (66). Research has also shown that CD4^+^ T cells isolated from ITP patients express more AhR (65). Furthermore, a raised level of IL-22 can be detected in ITP. An increase in the plasma concentration of IL-22 also has a positive relationship with Th22 cells, Th17 cells, and Th1 cells. As a result, these types of T cells work together, contributing to disease progression (67). Granzyme B, a cytolytic protein, is usually expressed by CD8^+^ T cells and natural killer (NK) cells. However, it is also expressed in Th22 cells, which may be one of the mechanisms by which Th22 cells play a role in ITP (68). Abnormal polarization of Th22 cells and Th1 cells with the aberrant secretion of IL-22 provide new targets for ITP therapy. After high-dose dexamethasone (HD-DXM) treatment, the concentration of IL-22 decreased, and the imbalance between Th1 and Th22 subpopulations was corrected. Additionally, the IL-22 level and Th1 and Th22 cells are positively correlated both before and after treatment. HD-DXM may correct the secretion of IL-22 by normalizing the polarization of Th22 cells and Th1 cells. Therefore, this treatment can be used to relieve symptoms of ITP (69).

### Abnormal Th9 cells

Th9 cells are an independent subpopulation of Th cells. When stimulated by TGF-β and IL-4, naïve T cells undergo differentiation and become Th9 cells, which secrete IL-9. Many typical transcription factors are not expressed in Th9 cells. However, Th9 cells typically express transcription factors including signal transducer and activator of transcription 6(STAT6), PU.1, interferon response factor 4 (IRF4), and BATF. Since Th9 cells participate in a range of immune responses through the secretion of IL-9, some autoimmune diseases, including ITP, may be associated with Th9 cells (70). Some findings have shown that the expression of IL-9 and the related transcription factors PU.1, IRF4, and BATF significantly rises in patients with active ITP. As the symptoms slowly subside, their expression levels gradually return to normal. Also, levels of Th9 cells and IL-9 are connected to the levels of Th17 cells and IL-17, suggesting that there is a positive correlation between these factors. In addition, IRF4 or BATF is found to be positively correlated with RORγ (71). Research indicates that IL-9 is mainly from Th17 cells and can lead to inflammatory diseases (72); however, according to another study, Th17 cells pathogenicity can be inhibited by IL-9 (73). Therefore, the specific mechanisms involved need further study. Overall, there is evidence that with increasing numbers of Th9 cells, the count of platelets increases accordingly (74), and the abnormal elevation of Th9 cells and IL-9 levels can induce ITP. In the future, targeted therapy against Th9 cells and IL-9 may be a new approach for treating ITP.

### Increased number and hyperfunction of Tfh cells

Tfh cells are the major T cell subpopulation in secondary lymphoid organs and spleen. The germinal centers (GCs) of secondary follicles in these organs are home to Tfh cells. They control how B cells develop, including the differentiation of B cells, immunoglobulin class switching, and the secretion of antibodies. It has been found that the spleens of ITP patients have significantly more Tfh cells compared to healthy controls, resulting in increased GCs and plasma cells (75). Additionally, Tfh cells in the peripheral blood and the number of platelets are negatively correlated in ITP. This finding suggests that Tfh cells may be an essential factor in causing ITP. IL-21 is a cytokine that is important for the formation of GCs and the development of Tfh cells. Tfh cells can produce large quantities of IL-21 and directly support CD8^+^ T cell function. In chronic ITP, IL-21 increases and significantly decreases after treatment (76). As a group of transcription factors with a leucine zipper, the AP-1 family controls gene expression in response to a range of external signals and regulates cellular processes, such as proliferation, differentiation, and apoptosis. c-Maf belongs to the AP-1 family and has a high expression in mature Tfh cells. There is a close relationship between the expression level of c-Maf and the development of B cells (77). It has been shown that c-Maf significantly elevates during chronic ITP and decreases after treatment (76). Bcl-6, a crucial gene for the differentiation and function of Tfh cells, has been confirmed to act synergistically with c-Maf (76). The deficiency of this gene leads to abnormal Tfh cell development, not only *in vitro* but also *in vivo*. Furthermore, the expression of Bcl-6 is required for GC responses (78). These results further confirm that Tfh cells play a part in ITP, whereby IL-21 induces Tfh cells to begin proliferating in large numbers, which in turn causes B cells to be activated and become plasma cells. These cells can produce antiplatelet antibodies. In addition, C-X-C motif chemokine ligand 13 (CXCL13) plays an important role in the immune microenvironment by binding to the chemokine receptor CXCR5. A study has shown that elevated levels of CXCL13 in ITP promote Tfh cell expansion by interacting with CXCR5 expressed on the surface of Tfh cells. The increased Tfh cells cause a further increase in CXCL13, which in turn induces B-cell proliferation and differentiation and the production of platelet-specific antibodies (79). Therefore, CXCL13 is a biomarker associated with ITP and a potential therapeutic target (80). The relationship between ITP and Tfh cells also provides pathways to be targeted for ITP therapy. For example, after the treatment with intravenous immunoglobulin, corticosteroids, or combination therapy, the number of Tfh cells decreases to normal levels and ITP symptoms are alleviated (76).

**Figure 2:**
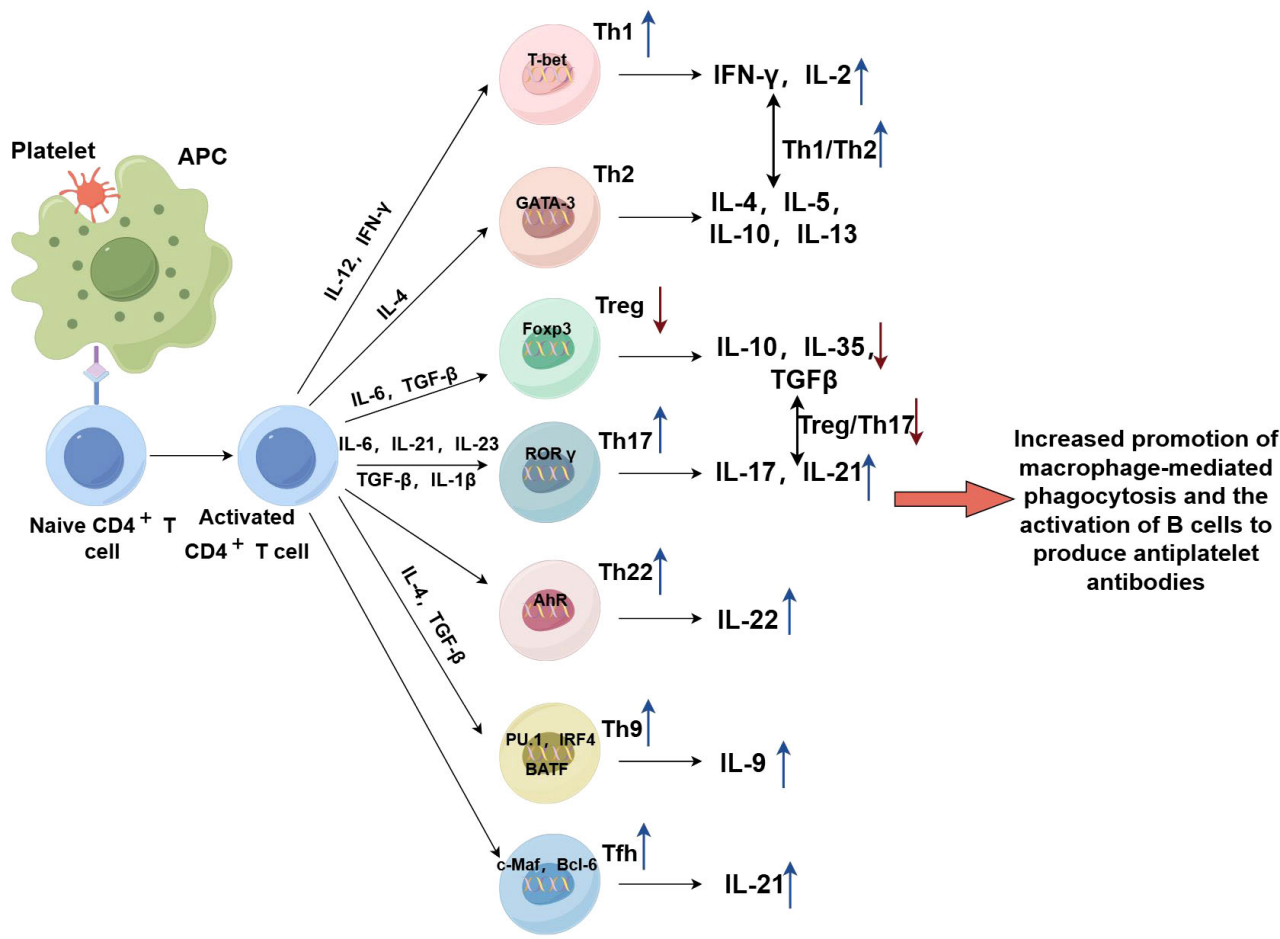
By interacting with antigen-presenting cells, naïve CD4 ^+^ T cells differentiate into functionally distinct activated CD4 ^+^ T cells that secrete different cytokines to play specific roles in the immune response against platelets. Dysregulated CD4^+^ T cell-mediated immunity is a hallmark of ITP pathogenesis. Th1, Th9, Th17, and Th22 cells collectively promote the phagocytic activity of macrophages, the activation of auto-reactive B cells, and the cytotoxicity of CTLs. The hyperfunction of Tfh cells causes an increase in the size of GCs and the number of plasma cells further facilitating the secretion of anti-platelet antibodies. The reduction of Tregs reduces the control of overreaction, limits self-tolerance and enhances autoimmune responses, which in turn contributes to an increase in the production of antiplatelet antibodies.

In summary, the dysfunctional proliferation of autoreactive T cells is responsible for the deficiency of auto-platelet antigen tolerance in ITP. Th1, Th17 and Th22 cells collectively promote: the function of macrophages, the activation of auto-reactive B cells, and the toxicity of T cells (81). The hyperfunction of Tfh cells causes an increase in GCs and plasma cells, which in turn increases the secretion of anti-platelet antibodies. The reduction of Tregs reduces the autoimmune tolerance and enhances autoimmune responses, which in turn causes more production of antiplatelet antibodies. In all, dysregulated CD4^+^ T cell-mediated immunity is an essential factor in the pathogenesis of this disease (**Figure 2**) (By Figdraw).

## Dysregulation of CD8^+^ T cells in ITP

As previously described, after platelet-specific autoreactive T cells are activated, they induce B cells to produce platelet-reactive antibodies (82). Macrophages in the spleen phagocytose antibody-coated platelets, after which they process and present GPIIb/IIIa autoantigens to GPIIb/IIIa-reactive CD4^+^ T cells, which causes more production of anti-platelet autoantibodies by B cells, creating various cycles of platelet destruction (83). However, a study demonstrated that by using monoclonal antibody-specific immobilization of platelet antigens (MAIPA), only approximately 50% of patients with ITP had anti-GPIb/IX or anti-GPIIb/IIIa antibodies (84). This is partly due to the limitations of the testing methods. However, excluding the limitations of the assay, the mechanism of non-antibody-mediated platelet destruction, such as through CD8^+^ T cells may also be the reason why antibodies cannot be detected on the platelet surface in some ITP patients. Some studies have revealed that CD8^+^ T cells increase the destruction of platelets in ITP patients, confirming the role of CD8^+^ T cells in ITP (85, 86). Furthermore, CD8^+^ T cell responses of ITP patients are biased toward Tc1 in the peripheral blood and spleen. Unlike normal controls, ITP patients have a greater Tc1/Tc2 ratio, suggesting that CD8^+^ T cells in ITP polarize toward type I (Tc1), thus accompanying the high level of IFN-γ and TNF-α, which leads to a pro-inflammatory state (27). At the same time, the autoimmune response cannot be counteracted due to the reduction of Tregs in the spleen, peripheral blood, and bone marrow (87). Therefore, dysregulation of CD8 ^+^ T cells may also be a main factor contributing to ITP.

**Figure 3:**
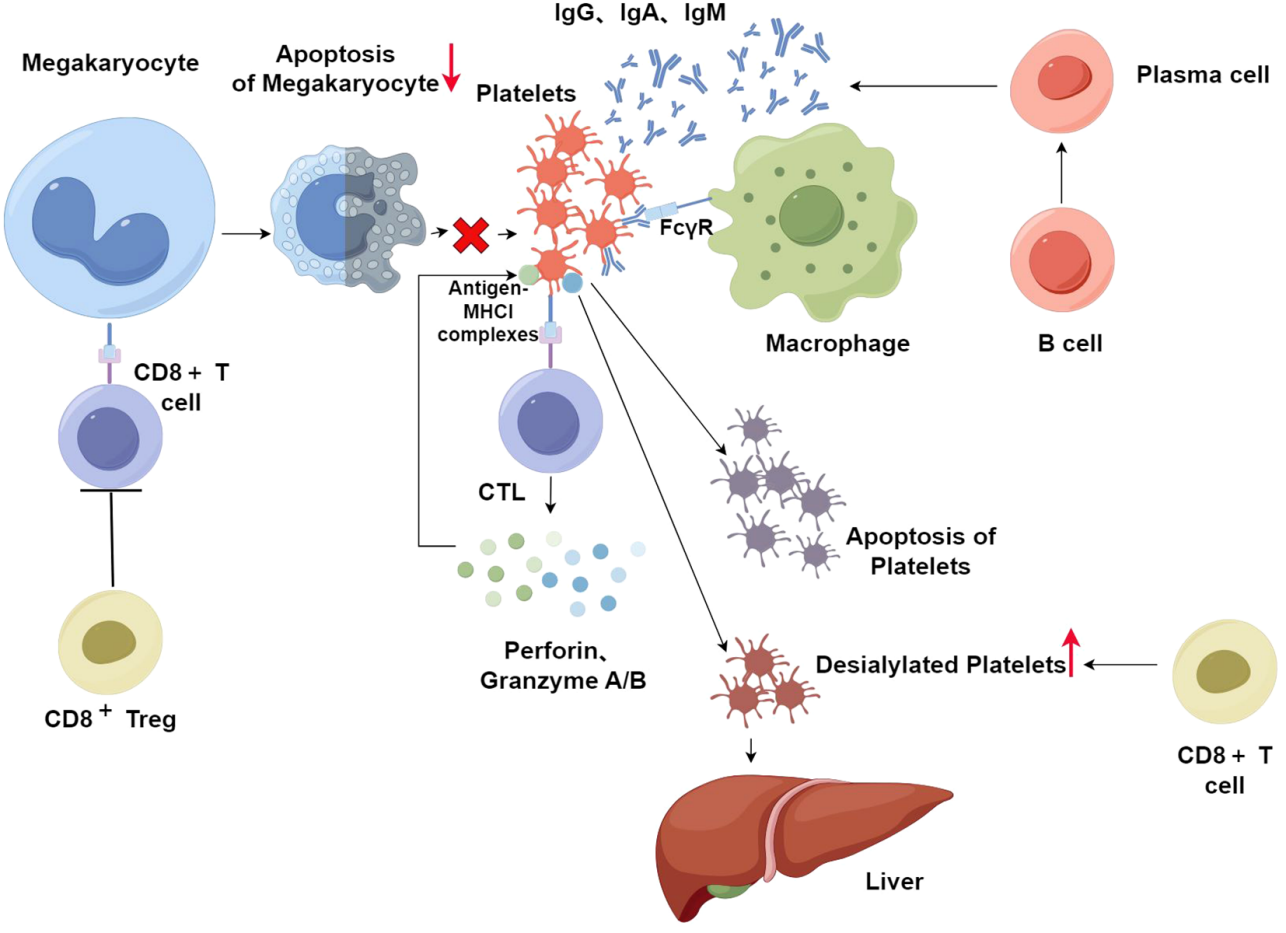
After platelet-specific autoreactive T cells are activated, they can recognize antigens on platelets and then induce B cells to produce platelet-reactive antibodies. After antiplatelet autoantibodies bind to platelets, macrophages phagocytose these antibody-coated platelets in the spleen. However, platelet-associated autoantibodies are not detected in all ITP patients. The excessive destruction of platelets in these patients is primarily through CD8^+^T cells. Platelets can express antigen-MHCI complexes on their surface, which in turn are recognized by CTLs. Then, CTLs secrete perforin, granzyme A, and granzyme B, which consequently destroy platelets via cytotoxicity. In addition, platelets in the circulatory system undergo desialylation and then clearance in the liver. CD8^+^ T cells potentiate the desialylation of platelets, which in turn leads to more platelets being cleared in the liver. CD8^+^ T cells also decrease platelets by preventing the apoptosis of megakaryocytes and increasing dysfunctional megakaryocytes, leading to suppression of platelet production. CD8^+^ Tregs suppress the development of CD4^+^ T cells and the cytotoxicity of CTLs. In ITP, CD8^+^ Tregs are decreased which can increase the destruction of platelets.

There are different mechanisms related to CD8^+^ T cells. Although platelets cannot experience the fragmentation of DNA (a hallmark of apoptosis in eukaryotes) because they are anucleate, platelets do experience other processes involved in apoptosis, including the activation of cysteine asparaginase, the depolarization of the mitochondrial membrane (ΔΨm), and exposure of phosphatidylserine (PS). Platelets express antigen-MHCI complexes on their surface (88). Therefore, platelets can be recognized by CTLs. Then CTLs can be activated and release interferon γ, and cause platelet apoptosis through TCR-mediated release of cytotoxic granules (89). In ITP patients, perforin, granzyme A, granzyme B, and Fas/Fas-L are overexpressed in CTLs. This indicates the abnormal activation of CTLs in ITP patients, which in turn directly destroys platelets through cytotoxic effects (86). Another study showed that the killer cell immunoglobulin-like receptor (KIR) down-regulates the role of CTLs and NKs through binding to MHCI, thereby preventing the lysis of the target cell. The expression of several members of the KIR family is greater in patients whose syndromes are in remission than in those with active disease, suggesting that the destruction of platelets by CTL-mediated cytotoxicity is inhibited by the upregulation of KIR, which in turn alleviates symptoms (85). In conclusion, CTLs enhance platelet destruction through cytotoxic effects, which in turn cause ITP. In addition, studies have demonstrated that platelets in the circulatory system undergo desialylation. The Ashwell-Morell receptor (AMR) identifies these desialylated platelets, which then undergo clearance in the liver. This clearance mechanism drives the JAK2-STAT3 signaling pathway in hepatic cells to activate, which primes the synthesis of thrombopoietin (TPO), thereby regulating the formation of platelets (90). Several experimental results suggest that in patients with active ITP, CD8^+^ T cells have the effect of potentiating the desialylation of platelets, which in turn leads to more platelets being cleared in the liver (91). In addition, it has been shown that mature myeloid megakaryocytes (MK) are capable of presenting endogenous antigens to CD8^+^ T cells *in vivo* via MHCI, resulting in reduced formation of platelets (92). Another mechanism is related to platelet production by megakaryocyte apoptosis. The role of megakaryocyte apoptosis in platelet production has been controversial. A study has suggested that both pro-survival and pro-apoptotic factors play a role in this process. The balance between the two factors is important in platelet production (93). When the balance is disturbed, for example, when CD8^+^ T cells cause inhibition of megakaryocyte apoptosis, this leads to an increase in dysfunctional megakaryocytes, which in turn causes inhibition of megakaryocyte maturation and platelet production (94). Abnormalities of CD8^+^ Tregs may also contribute to ITP. It has been demonstrated that CD8^+^ Tregs are very important for immunosuppression in a mouse model of ITP. Splenocyte-transplanted mice depleted of CD8^+^ T cells develop more severe ITP, while the injection of CD8^+^ T cells ameliorates symptoms, suggesting an overall protective function of CD8^+^ T cells, which is attributed to the presence of a subpopulation of CD8^+^ Tregs that have immunomodulatory functions. Experiments *in vitro* have shown that CD8^+^ Tregs suppress the development of CD4^+^ T cells and the cytotoxicity of CTLs (95). Therefore, a decrease in CD8^+^ Tregs is a crucial factor in causing ITP (**Figure 3**) (By Figdraw).

Studies on CD8^+^ T cells are providing new targets for ITP therapy. Decitabine, which is a cytidine antimetabolite analog, inhibits platelet apoptosis mediated by CD8^+^ T cells (96). The main mechanism is that low-dose decitabine inhibits the CTL-mediated cytotoxicity to autologous platelets via the PD-1 pathway, which in turn prevents the destructive effects of CTLs on platelets (97). Some findings suggest that IL-27 reduces the expression of granzyme B to inhibit CTL-mediated toxicity in ITP, which is induced by the reduced expression of T-bet. Therefore, IL-27 may be applied in ITP treatment in the future (98). In addition, we can learn from these studies that the detection of the number of CD8^+^ T cells helps to determine the diagnosis and outcome of treatments in ITP patients. Patients with more CD8^+^ T cells or a lower CD4^+^ T/CD8^+^ T cell ratio before treatment are less sensitive to the first-line therapy, making the level of CD8^+^ T cells a useful prognostic indicator for patients with ITP, especially the elderly (99).

## Genetic factors in ITP

A study based on microarray genetics has shown distinctive differences in gene expression profiles between healthy individuals and ITP patients (100). For example, the expression of two genes, TLR4 and S100A8, was significantly upregulated in chronic ITP patients compared to normal people. This study confirms the role of genetic factors in ITP. Another study has shown that the susceptibility of patients with ITP to cortisol therapy may also be genetically related. Due to this difference in sensitivity, the long-term efficacy of high-dose dexamethasone (HDD) at 6 months is approximately 54%, and that of prednisolone is about 43% (101). Genetic analysis revealed that genetic factors are mainly related to cytokine genes (102, 103) and T-cell co-stimulatory molecule genes. The specific role of genetic factors in the disease is not fully understood, but, they are certainly important for the development, progression, and treatment of ITP. This can help screen high-risk populations as well as for the prevention and treatment of ITP.

CD28, DNAM1 (CD226), and ICOS belong to the co-stimulatory molecules of T cells, while the co-suppressive molecules consist of CTLA4, PD1, and LAG3. Single nucleotide polymorphisms (SNPs) associated with these co-stimulatory and co-inhibitory molecules have been genotyped. A study has shown that CD28 rs1980422 increases the risk of ITP; the T allele of DNAM1 rs763361 is strongly related to cortisol resistance, and the T allele of rs6726035 in ICOS alleviates cortisol resistance. The T allele of rs36084323 in PD1 is closely related to the severity of ITP, where the TT/CT genotype significantly increases the likelihood of intractable ITP. However, the T allele of LAG3 rs870849 can reduce disease severity (104). Both the CTLA-4 rs11571316 G allele and rs3087243 G allele are linked to the low expression of CTLA-4. Because the CTLA4 gene is responsible for regulating the function of Tregs, the decreased expression of CTLA4 makes T-cells overactivated, which in turn leads to an increased risk of ITP accompanied by increased hemorrhage potential and lower platelets (105).

Analysis of T cell-associated inflammatory cytokine genes has shown that polymorphism in the IFN gene is closely associated with the generation of IFN-γ. The level of IFN-γ in the TT genotype of IFN-γ rs2430561 is higher than the AA genotype. In chronic ITP patients, the frequency of the TT genotype is greater than that in the healthy population (106), although this finding varies across different racial groups. Furthermore, it was observed that patients with a non-AA genotype experience a more significant decrease in platelet count, and they also exhibit a higher Th1/Th2 ratio (23). Therefore, polymorphisms in the IFN gene may be very important for the development and severity of the illness. The IL-17F gene is considered the gene that primarily regulates the activity of Th17 cells. The IL-17F rs763780 is associated with ITP susceptibility, and the C allele is less common in ITP patients. Furthermore, patients with the TT genotype have fewer platelets than patients with the TC genotype (107), suggesting that the C allele may prevent the development of ITP. As an inflammation-related gene, the expression of the caspase recruitment domain-containing protein 9 (CARD9) gene stimulates naïve T cells to develop into Th1 cells, which generate IFN-γ, and/or Th17 cells that produce IL-17 (108). SNPs in the CARD9 gene may alter the Th1/Th2 ratio and change the secretion of inflammatory cytokines, which in turn cause autoimmune diseases including ITP (109). The genetic analysis results confirmed that the CARD9 rs4077515 variant is linked to a decrease in both the susceptibility to and severity of ITP. This association might be due to the fact that the SNP rs4077515 promotes Th2 cell differentiation, re-establishes the balance between Th1 and Th2 cells, and helps ITP patients regain a state of immune tolerance, thereby reducing both the risk and severity of ITP (110).

## Discussion

Previous studies have demonstrated that increased platelet destruction by autoantibodies is a part of the mechanism of ITP, leading to accelerated platelet clearance in the reticuloendothelial system and relatively impaired formation of platelets by megakaryocytes (4). As research progresses, more evidence has confirmed that ITP is also a T-cell disease. Dysregulated T cell numbers and function lead to the disruption of self-tolerance mechanisms, particularly defects in the activity of T regulatory cells, including Th1 polarization, the reduction of Tregs in number and function, the hyperfunction of Th17 and Th22 cells, and elevated Tfh cells in both the spleen and peripheral blood, all of which lead to an increase in antiplatelet autoantibodies and macrophage phagocytosis. Furthermore, CTL-mediated cytotoxicity is enhanced, leading to increased platelet destruction. The role of genetic factors associated with T cells in ITP is also gaining attention. Genes affect the susceptibility as well as the severity of the disease and are also closely related to the sensitivity to therapeutic drugs.

Currently, the final target of ITP treatment is to inhibit active hemorrhage and prevent fatal bleeding. The American Society of Hematology guidelines recommend observation, corticosteroids, and intravenous immunoglobulin (IVIG) as the first-line therapy. Second-line therapy includes thrombopoietin receptor agonists (TPO-RAs), splenectomy, rituximab, and immunosuppressive agents (111). Current research on T cell functions in the pathogenesis of ITP reveals new therapies that target T cells, which are potentially effective ways to cure this disease (112). Dexamethasone restores not only the balance between Th1 and Th2 cells but also the number of Th17 cells and Tregs to normal levels while restoring the levels of T cell subsets by promoting GATA3 and FOXp3 expression and suppressing RORγt to be expressed (113). Therefore, routine treatment of ITP with high-dose dexamethasone (HD-DXM) can lead to improvement of clinical symptoms (63). However, ITP is a heterogeneous disease with a complex pathogenesis. Clinically, ITP patients, especially chronic ITP patients, often exhibit poor responses to current treatments or have difficulty tolerating long-term drug therapy. Therefore, new individualized treatments need to be further explored to reduce recurrence and progression. Eltrombopag (ELT), which belongs to TPO-RAs, promotes the production of platelets by activating the thrombopoietin receptor (TPO-R). ELT is effective, well tolerated, and has fewer side effects in the majority of patients (114). ELT, as well as other TPO-RAs, such as avatrombopag (115), are currently used in the treatment of chronic or persistent ITP. New T-cell-based therapeutic approaches are also emerging. These approaches can also be effective in chronic or persistent patients. For example, normal mesenchymal stem cells (MSCs) can regulate the equilibrium between Th1 and Th17 cells, induce Tregs to differentiate, and regulate the production of corresponding cytokines. Studies have shown that MSC function is decreased in ITP with abnormal regulation of T cell subsets, which in turn causes an imbalance in T cells. At present, attempts have been made to use MSCs to treat ITP and have achieved good efficacy (116). In addition, cytotoxic T lymphocyte-associated antigen 4-immunoglobulin (CTLA4-Ig) can transform autoreactive T cells into incompetent T cells lacking immunoregulatory functions, and thus inhibit platelet destruction by autoreactive T cells. In most ITP patients, CTLA4-Ig establishes tolerance to platelet antigens via incompetent T cells. Therefore, inducing autoreactive T cells to become incompetent by CTLA4-Ig can be applied as a treatment for refractory ITP (117). In addition, the analysis of T-cell-related genetic testing can provide an important basis for identifying high-risk groups, the prevention of disease, and the prediction of severity, as well as help in the selection of therapeutic drugs and the discovery of new therapeutic targets. One study has analyzed TCR Vβ and TCR CDR3 DNA of CD4^+^T and CD8^+^T cells. Specific VDJ fragments appear more frequently in T cells from ITP patients than in those from normal individuals. Additionally, individualized TCR CDR3 sequences exist in different ITP patients. CD4^+^ T cells prefer TRBV12-4, TRBV6-4, etc., while it is more common for CD8^+^ T cells to use TRBV12-4, TRBV6-4, and others (2). In the future, ITP patients will likely have access to individualized therapies targeting specific TCRs, such as CAR-T therapy.

The pathogenesis of ITP is complex. Although autoantibody-mediated platelet destruction is thought to be very important, more and more evidence suggests that T-cell-mediated immune abnormalities are equally crucial in causing ITP. There are still many mechanisms that are not fully understood, and in the future, as research progresses, it will further deepen the understanding of the disease and thus provide more effective ways to cure it.

## Glossary

ITP: Immune thrombocytopenia
IL: Interleukin
HRQOL: Health-related quality of life
GPs: Glycoproteins
FcγR: Fcγ receptor
Th cells: T helper cells,
Tfh cells: T follicular helper cells
Tregs: Regulatory T cells
CTL: Cytotoxic T lymphocyte
MHC: Major histocompatibility Complex
PBMCs: Peripheral blood mononuclear cells
IFN: Interferon
CTLA-4: Cytotoxic T-lymphocyte-associated antigen-4
GITR: Glucocorticoid-induced tumor necrosis factor receptor
TGF-β: Transforming growth factor-β
ROR γ: Receptor-related orphan receptor γ
TCR: T-cell receptor
ITAMs: Immunoreceptor tyrosine-based activation motifs
APCs: Antigen-presenting cells
HD-DXM: High-dose dexamethasone
AhR: Aryl hydrocarbon Receptor
NK cells: Natural killer cells
STAT6: Signal transducer and activator of transcription 6
IRF4: Interferon response factor 4
BATF: Basic leucine zipper ATF-like transcription factor
GCs: Germinal centers
HDACi: Histone deacetylase inhibitors
CXCL13: C-X-C motif chemokine ligand 13
MAIPA: Monoclonal Antibody-Specific Immobilization of Platelet Antigens
PS: Phosphatidylserine
KIR: Killer cell immunoglobulin-like receptor
AMR: Ashwell-Morell receptor
TPO: Thrombopoietin
HDD: High-dose dexamethasone
SNPs: Single nucleotide polymorphisms
CTLA4: Cytotoxic T lymphocyte-associated antigen 4
CTLA4-Ig: Cytotoxic T lymphocyte-associated antigen 4-immunoglobulin
CARD9: Caspase recruitment domain-containing protein 9
IVIG: Intravenous immunoglobulin
TPO-RAs: Thrombopoietin receptor agonists
MSCs: Mesenchymal stem cells
CAR-T: Chimeric antigen receptor T-cell immunotherapy
